# The impact of antioxidant-ciprofloxacin combinations on the evolution of antibiotic resistance in *Pseudomonas aeruginosa* biofilms

**DOI:** 10.1038/s41522-024-00640-3

**Published:** 2024-12-30

**Authors:** Doaa Higazy, Marwa N. Ahmed, Oana Ciofu

**Affiliations:** 1 Costerton Biofilm Center, Department of Immunology and Microbiology, University of Copenhagen, Copenhagen, 2200 Denmark; 2 Department of Microbiology, Faculty of Agriculture, Cairo University, Giza, 12613 Egypt

**Keywords:** Evolution, Biofilms, Antimicrobials

## Abstract

The evolution of antimicrobial resistance (AMR) in biofilms, driven by mechanisms like oxidative stress, is a major challenge. This study investigates whether antioxidants (AOs) such as N-acetyl-cysteine (NAC) and Edaravone (ED) can reduce AMR in *Pseudomonas aeruginosa* biofilms exposed to sub-inhibitory concentrations of ciprofloxacin (CIP). In vitro experimental evolution studies were conducted using flow cells and glass beads biofilm models. Results showed that combining CIP with antioxidants (CIP-AOs) effectively reduced the development of CIP resistance. Isolates from biofilms treated with CIP-AO had significantly lower minimum inhibitory concentrations (MICs) of CIP compared to those treated with CIP alone. Whole-genome sequencing (WGS) revealed mutations in the negative regulators of efflux pumps, *nfxB*, and *nalC*, in CIP-only treated biofilm populations. The occurrence of *nfxB* mutations was significantly lower in flow cell biofilms treated with CIP-AO compared to CIP alone. These findings suggest that antioxidants could play a role in mitigating AMR development in biofilms.

## Introduction

Evolutionary medicine emphasizes the importance of shifting the focus from merely developing new antibiotics, which inevitably leads to resistance, to addressing the core issue: the evolutionary process itself. This involves identifying strategies to prevent resistance development. One such approach involves improving the utilization of existing drugs^1^.

Experimental evolution studies showed that biofilm growth promotes the development of antimicrobial resistance and tolerance through specific mechanisms that have been recently reviewed^2^. One such mechanism is increased oxidative stress in biofilms^3^, leading to increased mutagenesis^3^. Moreover, treating bacteria with bactericidal antibiotics like CIP at sub-inhibitory concentrations increases the production of reactive oxygen species. This generates superoxide (O2^-^) and hydroxyl (OH^-^) radicals by inhibiting bacterial DNA gyrase and promoting the Fenton reaction, thereby increasing mutagenesis^4^. Bacteria employ antioxidant systems like catalases to defend against endogenous ROS by breaking down hydrogen peroxide (H_2_O_2_) into water (H_2_O) and oxygen (O_2_).

Transcriptomic studies on *Pseudomonas aeruginosa* under oxidative stress reveal that exposure to oxidizing agents like H_2_O_2_, NaOCl, and peracetic acid commonly results in the upregulation of detoxifying enzymes (e.g., catalase, superoxide dismutase), heat shock proteins, and virulence genes. Additionally, these studies show downregulation in genes related to primary metabolic processes, energy metabolism, and ribosomal biogenesis, suggesting a shift in cellular resources to manage oxidative damage. The variations in gene expression responses are influenced by factors such as the type and concentration of oxidizing agents, bacterial strains, and growth phases during treatment, highlighting the adaptability and resilience of *P. aeruginosa* under different oxidative conditions^5,6^.

However, in case of increased ROS production under stress conditions, such as biofilm growth or antibiotic treatment, the antioxidant mechanisms can be overwhelmed, leading to oxidative stress. We have shown previously that the evolution of antibiotic resistance in *P. aeruginosa* biofilms lacking the major catalase KatA (Δ*katA*), with impaired ability to scavenge ROS, was accelerated compared to WT PAO1 biofilms and Δ*katA* planktonic cultures^7^.

In addition to mutational resistance, biofilms also recruit mechanisms for adaptive resistance^8^. Mutational and adaptive antimicrobial resistance in biofilms can occur through stress responses such as “Save our souls” SOS, activated by DNA lesions caused by the CIP, either as a direct effect of the blocking of DNA gyrase or through ROS induction^4,8,9^.

Molecules, such as antioxidants (AOs), have been shown to reduce the stress-induced mutation rate in *Escherichia coli*. N-acetyl-cysteine was shown to block the SOS system and ROS production^10^. At the same time, Edaravone, a ROS scavenger, inhibits CIP-induced mutagenesis without affecting the SOS response^11^.

Both NAC and ED are approved for clinical use. NAC, which is a glutathione precursor, is used as a mucolytic agent and for the treatment of a range of disorders, including acetaminophen intoxication^12^. ED is registered for treating amyotrophic lateral sclerosis and stroke caused by cerebral thrombosis and embolism^13^. NAC does not inhibit the activity of anti-pseudomonal drugs, including CIP (except for imipenem)^10,14^.

Thiourea (THU), a ROS scavenger with a high affinity for hydroxyl radicals, has been previously shown to decrease ROS formation and the mutagenic effect of sub-inhibitory concentrations of antibiotics in planktonic *E.coli*^4^. We previously demonstrated that THU inhibits CIP-induced ROS in *P. aeruginosa* biofilms^15^. During CIP treatment of PAO1 biofilms, there was a significant increase in hydroxyl radical (OH•) production, contributing to the antibiotic’s bactericidal activity. The addition of THU reduced fluorescence signals from both hydroxyl radical-specific probes (HPF) and general ROS probes (DCHF-DA), indicating a decrease in ROS levels. This suggests that THU effectively neutralizes OH•, thereby mitigating the oxidative stress involved in CIP-induced bacterial killing^15^. Thiourea scavenges ROS, reducing oxidative stress and downregulating the transcription of error-prone DNA polymerase and MutM, which is part of the DNA oxidative repair system (GO), playing a role in mutagenesis. THU limits the accumulation of oxidative DNA damage, specifically 8-hydroxy-2′-deoxyguanosine (8-HOdG), thereby preventing mutations that contribute to antibiotic resistance^16^. We have previously shown that GO mutants of *P. aeruginosa* have increased mutation rates and exhibit antimicrobial resistance due to mutations in *nfxB*^17^. Antibiotic resistance in *P. aeruginosa* strains with increased mutation frequency arises due to inactivation of the DNA oxidative repair system^18^.

All these data suggest that oxidative stress plays an important role in the evolution of antimicrobial resistance. Planktonic *E. coli* has been investigated for the use of antioxidants as anti-mutagenic drugs^19,20^. The results showed the potential use of ED to decrease the occurrence of a hypermutable population (the “gambler population”)^19^ and inhibit the dissemination of plasmid-encoded resistance^20^.

The MexCD-OprJ efflux pump, part of the resistance-nodulation-cell division (RND) superfamily, is typically inactive in wild-type *P. aeruginosa* strains and does not contribute to its intrinsic antimicrobial resistance^21^. However, this pump can expel a wide range of substances, including antimicrobial agents, biocides, organic solvents, dyes, and detergents, and is controlled by repressors such as NfxB and EsrC, which regulate the expression of the mexCD-oprJ operon^22^. The *nfxB* gene, which encodes the NfxB repressor protein, is located upstream of the mexCD-oprJ structural genes, with an intergenic regulatory region enabling divergent transcription^23^. Mutations in the *nfxB* gene impair NfxB’s repressor function, leading to the overexpression of MexCD-OprJ; strains with such mutations are known as *nfxB*-type mutants. This study aimed to test the hypothesis that the two clinically approved antioxidants, NAC and ED, can function as anti-mutagenic agents to prevent the development of antimicrobial resistance in *P. aeruginosa* biofilms exposed to sub-inhibitory concentrations of CIP, with THU serving as a positive control. To address this hypothesis, experimental evolution studies were conducted in vitro using two *P. aeruginosa* biofilm models exposed to sub-inhibitory CIP alone or combined with one of the two antioxidants, CIP-AO. The anti-mutagenic effect was assessed using a construct where *gfp* expression indicates mutations in *nfxB*, the negative regulator of mexCD-oprJ. Here, we demonstrate that the occurrence of *nfxB* mutants was reduced in biofilms treated with one of the three combined treatments: CIP-THU, CIP-NAC, and CIP-ED.

## Results

### Treatment with antioxidants decreases the CIP-induced ROS formation in planktonic cultures

**Fig. 1:**
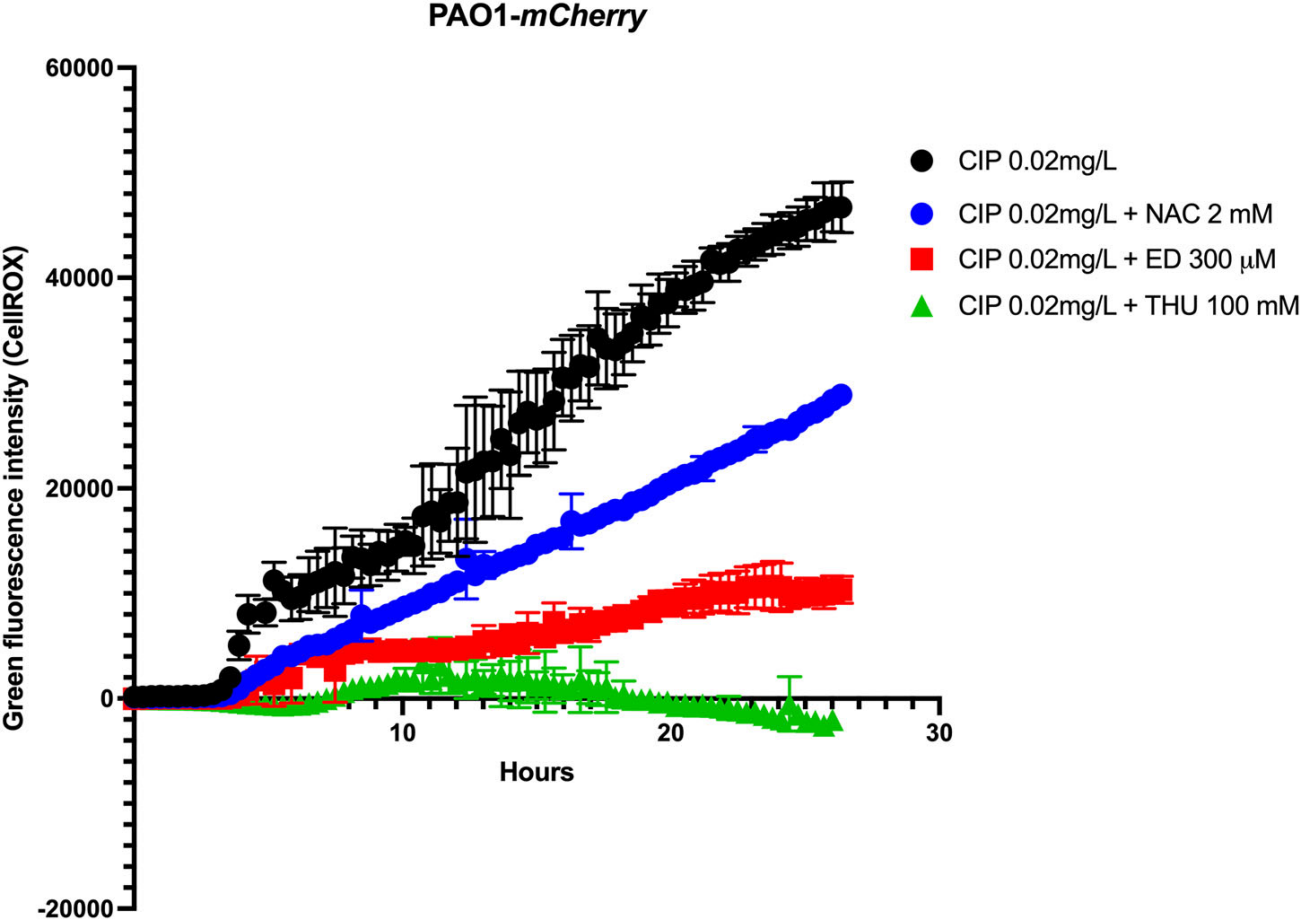
Monitoring ROS levels in planktonic cultures treated with ciprofloxacin and antioxidants. The green fluorescent signal of CellROX, used to monitor ROS levels during the planktonic growth of the mCherry-tagged PAO1 strain, was assessed under various treatment conditions. Bacterial cultures were exposed to Ciprofloxacin (CIP, black), CIP combined with N-acetyl-L-cysteine (CIP + NAC, blue), CIP with Edaravone (CIP + ED, red), and CIP with Thiourea (CIP + THU, green). All antioxidant (AO) treatments significantly reduced the green fluorescence signal compared to CIP alone (*p* = 0.0001). Among the antioxidant treatments, CIP + NAC and CIP + ED showed significantly higher green fluorescence signals compared to CIP + THU (*p* = 0.0001).

To investigate the potential of the different AOs to decrease the CIP-induced ROS formation, we visualized the ROS production using CellROX™ Green Reagent (ThermoFisher, US) during 24 h exposure of a planktonic culture of the mCherry tagged PAO1 strain to CIP (0.02 mg/L) or CIP-AO combinations (Edaravone, *N*-Acetyl-L-cysteine, and Thiourea) (Fig. 1). The ROS detector showed a decreased green fluorescence level in the cells treated with CIP + ED, CIP + THU, and CIP + NAC combinations (*p* < 0,0001) compared to those treated with CIP alone. NAC combined treatment exhibited higher ROS levels (green fluorescence) than both ED and THU, while THU showed the lowest ROS levels (*p* < 0.0001). To further validate the findings, the experiment was repeated using a different antibiotic, Tobramycin. Consistent with the results observed with CIP, the combination of Tobramycin with antioxidants significantly reduced the green fluorescence signal compared to Tobramycin treatment alone, indicating a decrease in ROS levels (Supplementary Fig. 1).

### Evolution of flow-cell biofilms treated with CIP or CIP-AO

Further, we tested if the ROS scavenger effect of AO, observed in CIP-treated cultures, led to an antimutagenic effect in the more complex environment of biofilms. The study relied on the reporter *P. aeruginosa* PAO1-*mCherry*-P_*CD*_-*gfp*+ strain. The fluorescent reporter strains were created by tagging wild-type PAO1 and an isogenic PAOΔ*nfxB* strain with *mCherry* at the chromosomal *attB* site, using a mini-CTX vector, ensuring constitutive expression under the P_*trc*_ promoter. A mini-Tn7 construct harboring *P*_*CD*_*-gfp+* was then integrated, enabling green fluorescence due to an enhanced GFP variant, which was previously described in detail^24^.

**Fig. 2:**
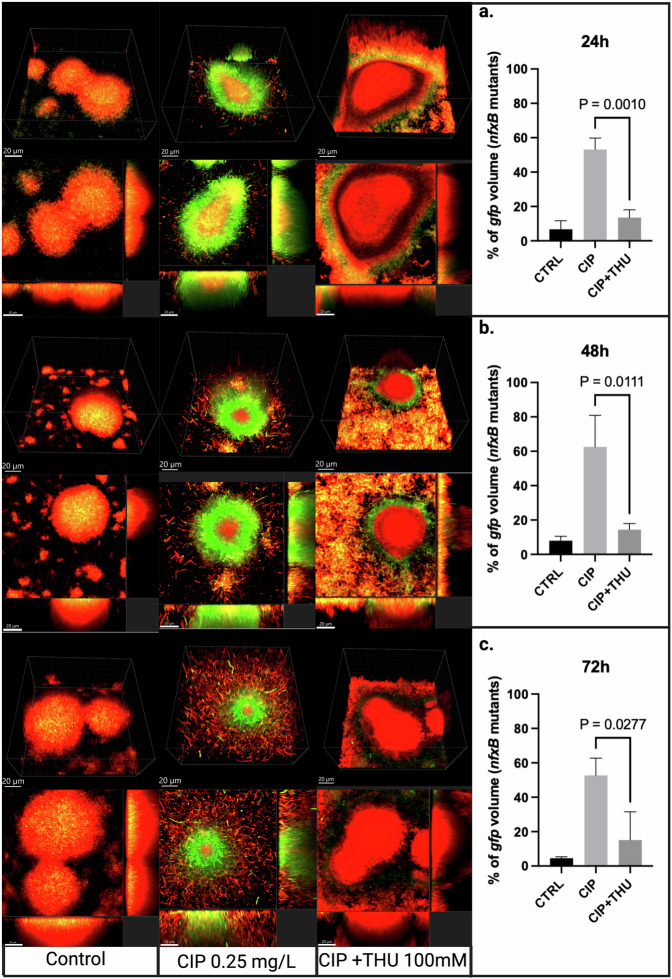
Biofilm structure and ROS response to CIP and CIP + THU treatments. Confocal laser scanning microscopy (CLSM) images of flow-cell biofilms at 24 hours **a**, 48 hours **b**, and 72 hours **c** under different treatment conditions: untreated control, ciprofloxacin (CIP, 0.25 mg/L), or a combination of CIP and 100 mM Thiourea (CIP + THU). Red fluorescence corresponds to wild-type cells mCherry constitutively, while the green fluorescence represents *nfxB* mutants expressing GFP via the *P*_*CD*_*-gfp* reporter. Z-stacks were acquired using a Zeiss 880 microscope and processed using Imaris 10.1 software (Bitplane). The images include orthogonal 3D views of the biofilm (upper) or sections (lower), with an overlay of fluorescence from the red and green channels. Quantification of the GFP % volume is shown in the left panel at each time point **a**–**c**. All microscopy images were captured under a scale bar of 20 µm.

The potential antimutagenic effect of AO on CIP-treated biofilms was investigated by CLSM microscopy of 72 h old biofilms of PAO1-*mCherry*-P_*CD*_-*gfp*+ which was either untreated (CTRL), treated with CIP 0.25 mg/L, or treated with both CIP and an antioxidant for three days. Biofilms treated with a combination of CIP and THU exhibited a substantial reduction in *nfxB* mutants compared to those treated solely with CIP, as represented by the decrease in the percentage of green GFP fluorescence. Specifically, the mean percentages were 13.5% and 53% (*p* = 0.001), respectively, 24 hours post-treatment (Fig. 2a). Likewise, at 48 hours and 72 hours, the percentage of *nfxB* mutants continued to be lower in the combined treatment group compared to the CIP treatment alone. Specifically, at 48 hours, the mean percentages were 14.3% and 62.5%, respectively, with a *p*-value of 0.0111 (Fig. 2b). Similarly, at 72 hours, the mean percentages were 15% and 52.6%, respectively, with a *p*-value of 0.0277 (Fig. 2c).

**Fig. 3:**
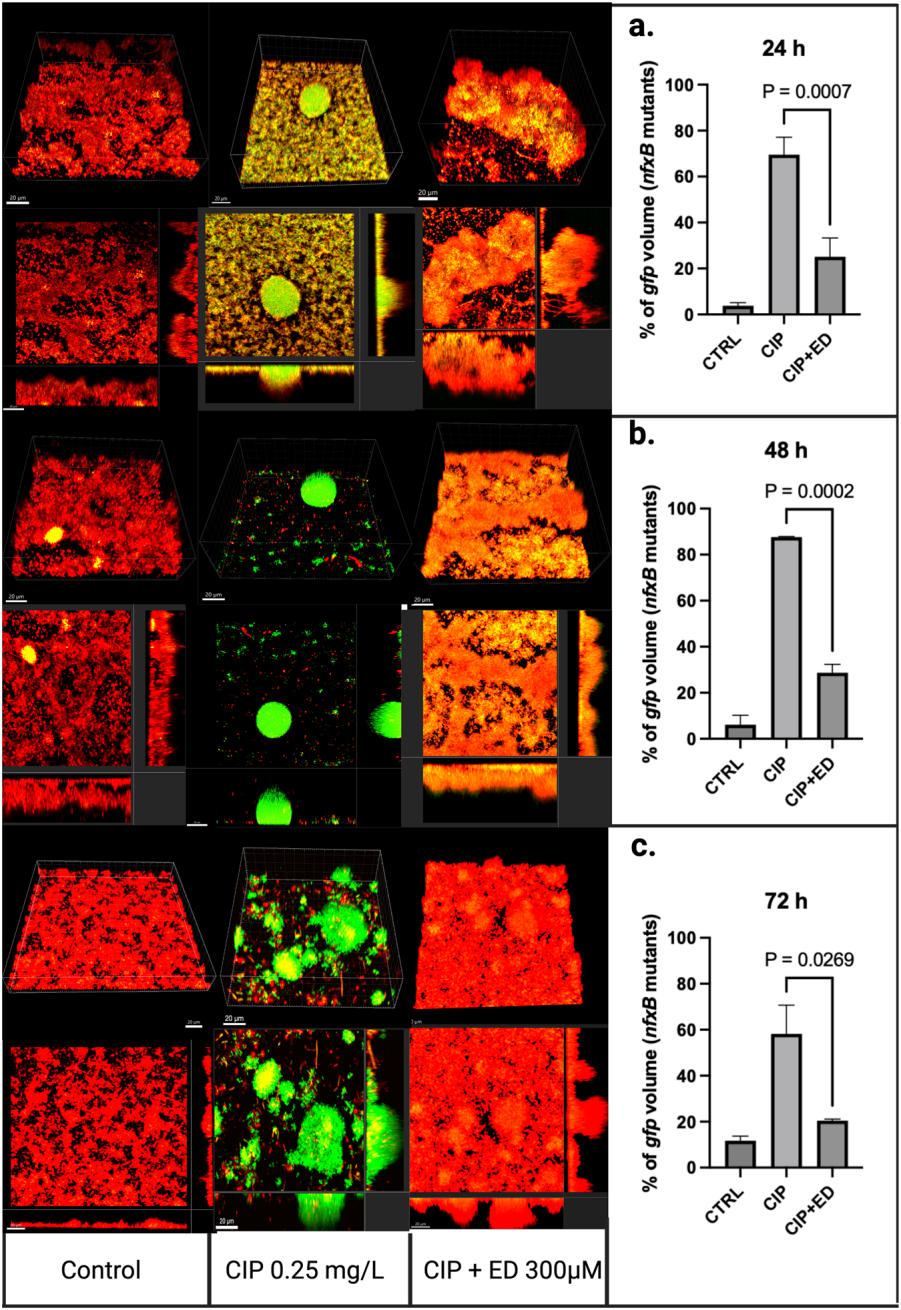
Biofilm structure and ROS response to CIP and CIP + ED treatments. CLSM images of flow-cell biofilms at 24 hours **a**, 48 hours **b**, and 72 hours **c** under different treatment conditions: untreated control, ciprofloxacin (CIP 0.25 m/L), or a combination of CIP and 300 µM Edaravone (CIP + ED). The red fluorescence corresponds to wild-type cells expressing mCherry constitutively, while the green fluorescence represents *nfxB* mutants expressing GFP via the *P*_*CD*_*-gfp* reporter. Z-stacks were acquired using a Zeiss 880 microscope and processed using Imaris 10.1 software (Bitplane). Images include orthogonal 3D views of the biofilm (upper) or sections (lower), with an overlay of fluorescence from the red and green channels. The quantification of the gfp % volume in the three conditions is shown in the left panel at each time point (a-c). All microscopy images were captured under a scale bar of 20 µm.

**Fig. 4:**
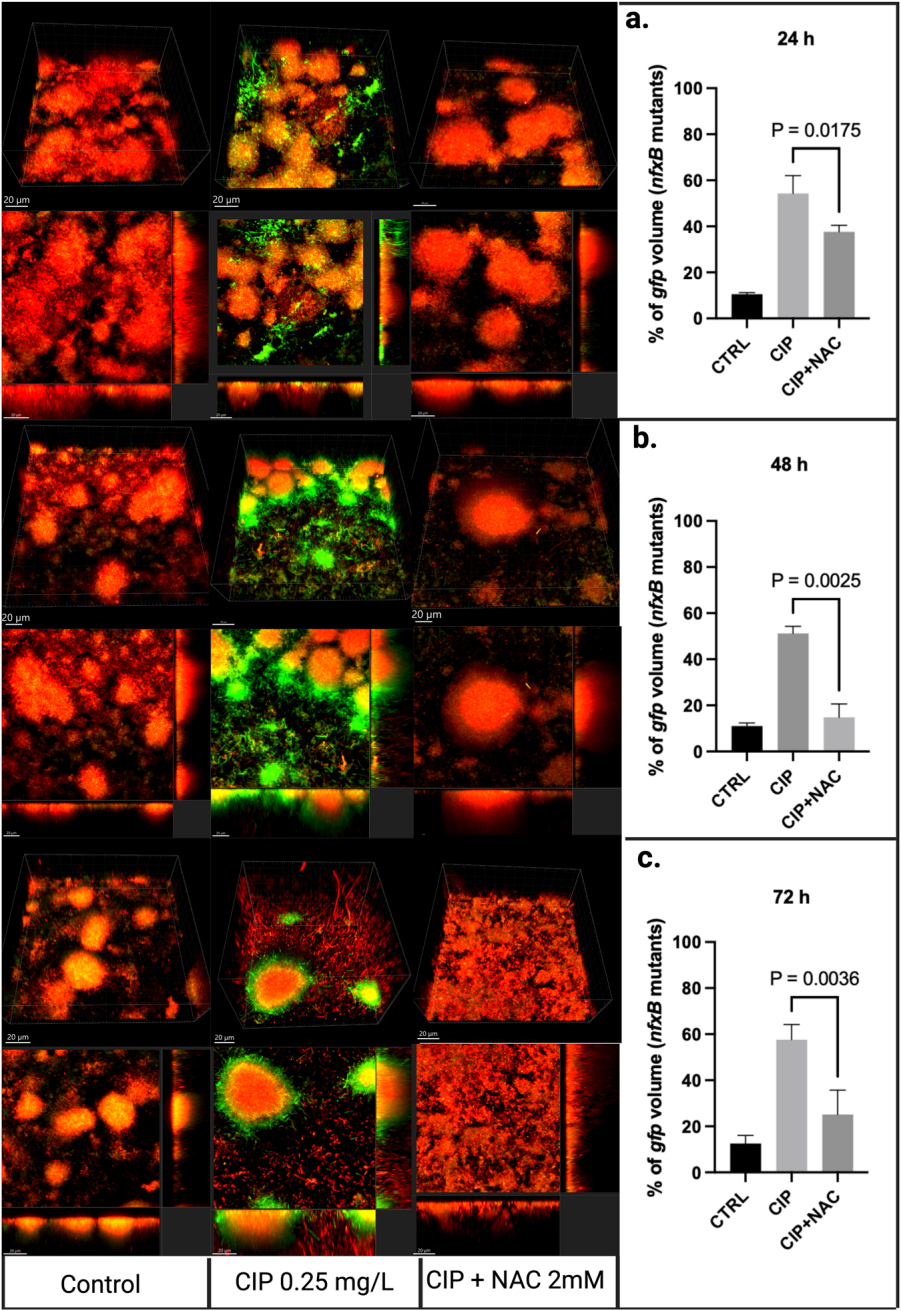
Biofilm structure and ROS response to CIP and CIP + NAC treatments. CLSM images of flow-cell biofilms at 24 hours **a**, 48 hours **b**, and 72 hours **c** under different treatment conditions: untreated control, ciprofloxacin (CIP 0.25 mg/L), or a combination of CIP and 2 mM N-acetyl-L-cysteine (CIP + NAC). Red fluorescence corresponds to wild-type cells expressing mCherry constitutively, while the green fluorescence represents *nfxB* mutants expressing GFP via the *P*_*CD*_*-gfp* reporter. Z-stacks were acquired using a Zeiss 880 microscope and processed using Imaris 10.1 software (Bitplane). The images include orthogonal 3D views of the biofilm (upper) or sections (lower), with an overlay of fluorescence from the red and green channels. The quantification of the gfp % volume in the three conditions is shown in the left panel at each time point **a**–**c**. All microscopy images were captured under a scale bar of 20 µm.

The same experiment was repeated using the antioxidants ED and NAC and showed similar results to thiourea in terms of GFP reduction with AO treatment. The *nfxB* mutation developed rapidly 24 hours following exposure to CIP in both experiments (Figs. 3, 4). Similarly, both antioxidants showed lower *nfxB* mutants for the CIP-antioxidant combined treatment. The CIP-ED combined treatment showed a mean percentage of 25.1% of *nfxB* mutants compared to 69.5% for CIP treatment (*p* = 0.0007) following 24 h of treatment (Fig. 3a), and it remained significantly lower than CIP at 48 h and 72 h following treatment (*p* = 0.0002, 0.0269, respectively) (Fig. 3b, c). NAC showed a similar trend with a substantially lower percentage of *nfxB* for the antioxidant-CIP combined treatment compared to the CIP treatment alone at the three different time points 24 h, 48 h, and 72 h (*p* = 0.0175, *p* = 0.0025, and *p* = 0.0036, respectively) (Fig. 4a–c).

The effect of antioxidants on biofilm formation was also assessed using the reporter strain PAO1-*mCherry*-P*CD*-*gfp* to determine whether they exhibit antimicrobial properties. The results indicated a slight increase in biofilm biomass in the three AO-treated groups compared to the untreated control. Among these, treatment with THU showed the most significant increase in biomass compared to the control (*P* = 0.0011) (Supplementary Fig. 2a,b).

### Population analysis profiles of flow-cell biofilm populations

**Fig. 5:**
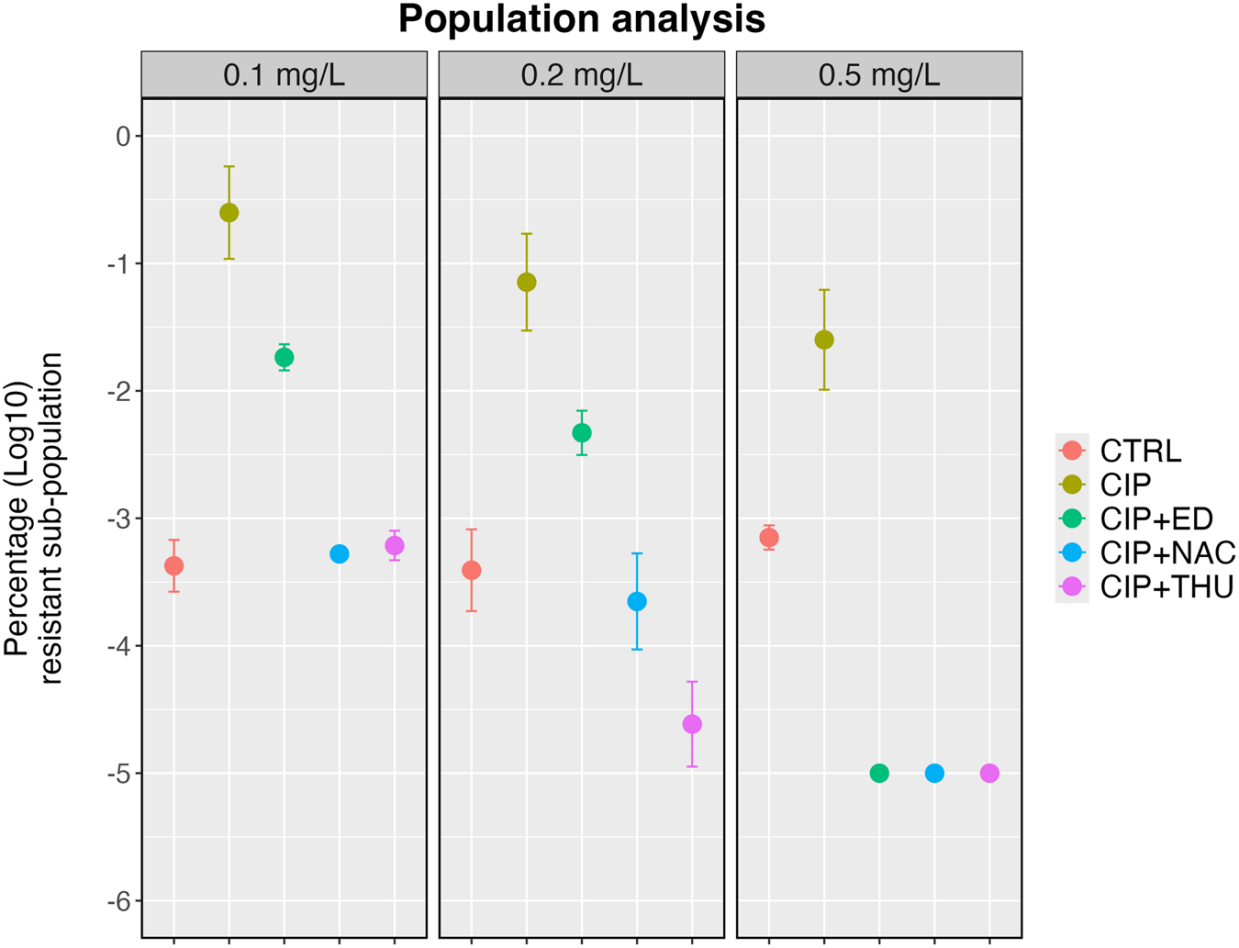
Population analysis in flow-cell biofilms. Population analysis of harvested bacteria from flow-cell biofilms after 72 hours of treatment. Biofilms were treated with ciprofloxacin (CIP), combination of CIP and Edaravone (CIP + ED), a combination of CIP and N-acetyl-L-cysteine (CIP + NAC), or a combination of CIP and Thiourea (CIP + THU), with untreated biofilms as a control (CTRL). The percentage of surviving bacteria at different CIP concentrations (0.1, 0.2, and 0.5 mg/L) is shown as log10 values. Statistical significance was determined using a t-test (*p < 0.05).

The populations harvested from different flow cell channels with different treatments were analyzed by population analysis profiles (PAP). The bacterial populations were plated on the LB agar supplemented with CIP at different concentrations (0.1, 0.2, and 0.5 mg/L) after passing twice on antibiotic-free media (Fig. 5). The results showed a decrease in the subpopulation grown on the different CIP concentrations for all the AO combined treatments compared to CIP, though not statistically significant for ED on 0.1 and 0.2 mg/L. Notably, a significant reduction was observed at 0.5 mg/L in populations exposed to the combined treatment (*P* = 0.000611).

### Population analysis of biofilm populations from glass-bead evolution

**Fig. 6:**
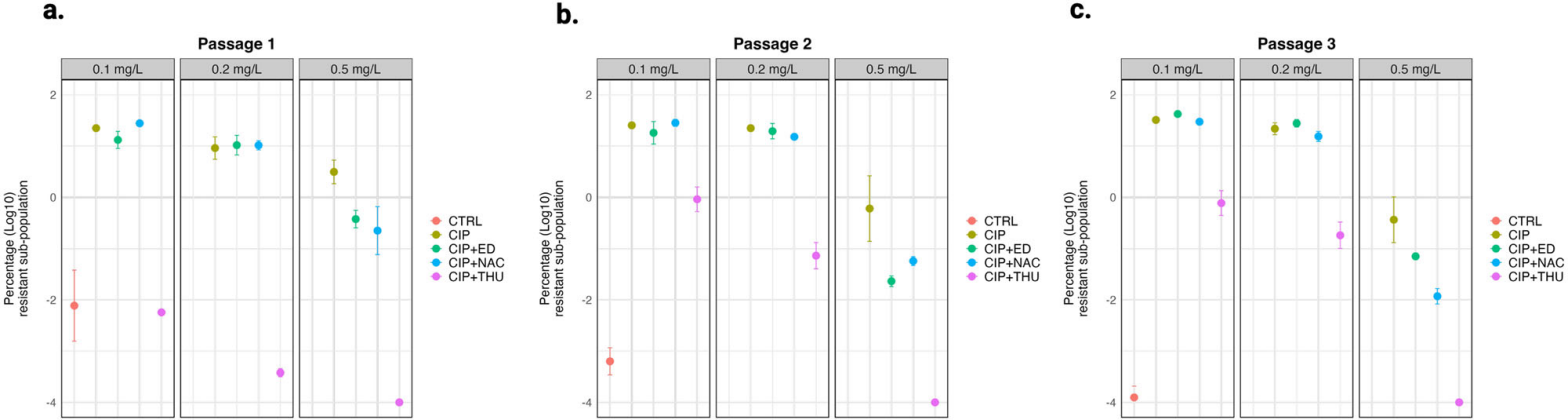
Population analysis of evolved populations in glass beads biofilm experiment. Population analysis of bacteria from glass bead biofilms after three different passages of treatment in an evolution experiment. Biofilms were treated with ciprofloxacin (CIP), a combination of CIP and Edaravone (CIP + ED), a combination of CIP and N-acetyl-L-cysteine (CIP + NAC), or a combination of CIP and Thiourea (CIP + THU), with untreated biofilms as a control (CTRL). The percentage of surviving bacteria at different CIP concentrations (0.1, 0.2, and 0.5 mg/L) is shown as log10 values for **a** passage1, **b** passage 2, and **c** passage 3. Statistical significance was determined using a t-test (*p < 0.05).

To validate the results obtained in flow cells in another biofilm model, we used the glass beads biofilms as a stationary biofilm model. Experimental evolution of *P. aeruginosa* surface-attached biofilms formed on glass beads was conducted for three passages under exposure to sub-MIC CIP and AOs-CIP combination treatments, as presented in (Supplementary Fig. 3). We performed population analysis profiles on the sonicated biofilm populations after passing them twice on an antibiotic-free medium (Fig. 6a–c). The population analysis showed a decrease in the resistant subpopulation only for the CIP-THU combined treatment, which was not significantly observed for the combined treatments with ED or NAC across the three different passages (Fig. 6a–c). Although the THU combined treatment PAP exhibited increased tolerance over passages, it remained significantly lower on CIP plates of 0.125 and 0.25 mg/L compared to the tolerant subpopulation obtained from CIP-treated cells. Which was further diminished on 0.5 mg/L CIP plates in the three different passages (*P* = 0.001).

### Population analysis of evolved planktonic cultures

**Fig. 7:**
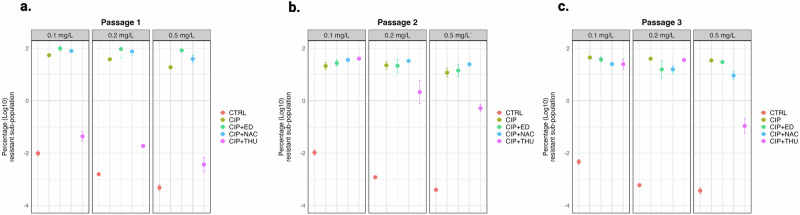
Population analysis for three different passages of planktonic evolution experiment. The planktonic populations have been exposed to either a ciprofloxacin concentration of 0.125 mg/L (CIP), a combination of CIP + ED (ED), a combination of CIP + NAC (NAC), or a combination of CIP + THU (THU) or untreated (CTRL). The results are presented as a log10 for the percentage of survived bacteria on plates with different CIP concentrations (0.1, 0.2, and 0.5 mg/L) for **a** passage 1, **b** passage 2, and **c** passage 3. (t-test, * *p* < 0.05).

We further investigated whether the ROS scavenging properties of AO could mitigate mutagenesis during evolution in planktonic cultures subjected to CIP treatment. Population analysis profiles were generated for planktonic cultures of PAO1-*mCherry*-P_*CD*_-*gfp*+ treated with either CIP alone or in combination with AO after each evolutionary passage (Fig. 7). Our results revealed no significant difference in the size of the resistant subpopulation between the CIP-treated group and the group treated with the combination of CIP and AO, except for the CIP-THU treatment in the initial passage. Interestingly, we observed that the filamentation of the *P. aeruginosa* cells treated with CIP in planktonic cultures was abolished by CIP-THU, which wasn’t observed for other AOs (Supplementary Fig. 4).

### MICs and growth rates of colonies recovered from the highest CIP concentration of PAP

**Fig. 8:**
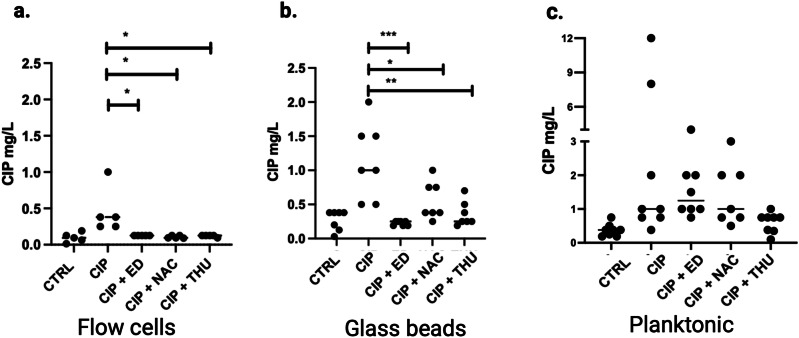
MICs of ciprofloxacin for evolved isolates. Minimal inhibitory concentrations (MICs) of ciprofloxacin for bacterial isolates collected from CIP-containing plates in the flow-cell biofilm populations (**a**), glass bead biofilm populations (**b**), and planktonic populations (**c**). Isolates were collected from untreated populations (CTRL), or populations treated with ciprofloxacin alone (CIP), or with combinations of CIP and Edaravone (CIP + ED), CIP and N-acetyl-L-cysteine (CIP + NAC), or CIP and Thiourea (CIP + THU).

Different isolates were collected from the plates with the highest CIP concentrations from the PAP, and their MICs were determined. The results showed significantly higher MIC levels for the isolates from CIP treatment compared to those from different AO combined treatments in both biofilm models (Fig. 8a,b). However, this trend wasn’t similar in the planktonic evolution MICs, which exhibited high variability among different isolates (Fig. 8c). To assess the fitness cost of CIP-resistant mutants isolates from PAP of planktonic and biofilm populations, growth curves in LB medium were performed, and growth rates were calculated. No significant differences were found between the growth rates of CIP-treated and control isolates, except for CIP-THU in planktonic and glass bead isolates (Supplementary Figure 5a–c).

### Genetic determination of mutations developed during the flow-cell experiments using different treatments

**Fig. 9:**
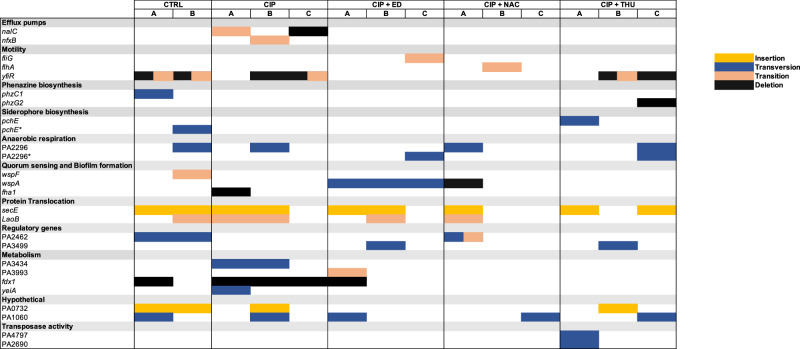
Genetic variations in flow-cell biofilm populations. Overview of genetic variations identified in populations from flow-cell biofilms after 72 hours of treatment. Populations included untreated controls (CTRL), populations treated with ciprofloxacin alone (CIP), or with combinations of CIP and Edaravone (CIP + ED), CIP and N-acetyl-L-cysteine (CIP + NAC), or CIP and Thiourea (CIP + THU). Each treatment group was represented by three sequenced populations, except the control, which had two. Genes with mutations at different positions but of the same type are marked with an asterisk (*). Mutations are categorized by their functional impact, with different types represented by distinct colors.

The WGS results from the different treated flow cell populations (Fig. 9) revealed CIP-treatment-specific mutants, such as a transition mutation in the negative regulator of *mexCD-oprJ nfxB* which explains the higher green fluorescence obtained in different CIP-treated channels compared to control and antioxidants-treated channels. In addition, there are two different mutations in the gene *nalC* (transition and deletion), which is a negative regulator of the MexAB-OprM efflux pump system in *P. aeruginosa*. Mutations in genes involved in flagella were observed in CIP+ED and CIP + NAC treated populations. The population CIP + ED gained a stop-mutation (transition) in the flagellar *fliG* gene, which could possibly disrupt flagellar function, impair bacterial motility and chemotaxis, attenuate virulence, and alter biofilm formation. The CIP + NAC treated population gained a stop-mutation (transition) in the flagellar assembly gene *flhA* which plays a crucial role in the type III secretion system (T3SS), which is responsible for the assembly and regulation of the flagellar apparatus. Interestingly, different types of mutations in *wspA* were observed in CIP + NAC (deletion) and CIP + ED (transversion) treated biofilms. In the CIP + ED treated biofilm population, a missense variant in the *wspA* gene which was presented in the three replicates was observed. CIP exhibits a notably higher NS/dS ratio compared to CTRL, indicating stronger positive selection and a greater tendency for protein-altering mutations. This suggests that CIP exerts significant selective pressure, promoting adaptive mutations that enhance bacterial survival in the antibiotic environment (Supplementary table 1).

## Discussion

Our results demonstrate that antioxidants such as ED, NAC, and THU could reduce the occurrence of *nfxB* mutants in CIP-treated *P. aeruginosa* biofilms, suggesting that decreasing oxidative stress in biofilms can prevent the evolution of resistance. However, the effect of these antioxidants in ROS-related mutagenesis exhibited variations depending on the specific antioxidant and the bacterial growth form (planktonic vs. biofilm and flow-cell biofilms vs. glass beads biofilm). The initial measurement of CIP-induced ROS formation using CellROX™ green reagent revealed that there was a significant decrease in cells treated with CIP combined with NAC, ED, or THU compared to CIP alone. NAC and ED showed less inhibitory effects on the CIP-induced ROS production than THU, indicating that not all antioxidants are equally effective in this context. This could be due to differences in the mechanisms by which these antioxidants neutralize ROS or interact with cellular components. While THU has a strong affinity for hydroxyl radicals formed under CIP treatment and ED has shown to be a broad inhibitor of ROS, NAC exhibits a poor antioxidant effect on CIP-induced ROS. However, it has been shown in *E.coli* planktonic cultures exposed to ½ MIC CIP that ROS production was inhibited by 40% after 8 h of exposure to NAC and the SOS response by 75%^10^. This discrepancy might be due to the different bacterial pathogens and methods of ROS detection.

The flow-cell biofilm experiments provided deeper insights into the antimutagenic potential of antioxidants. All the tested AOs ED, NAC, and THU significantly reduced the development of CIP-induced *nfxB* mutants in flow-cell biofilms, indicating their role in mitigating antibiotic resistance. These findings suggest that antioxidants can play an important role in limiting the emergence of resistant subpopulations in biofilm-associated infections and showing their potential antimutagenic effect in *P. aeruginosa* biofilms.

Population analysis profiles (PAP) from flow-cell biofilm populations and glass-bead biofilms confirmed the reduction in resistant subpopulations with AO-CIP combined treatments. However, this effect was more significant in flow-cell biofilm biofilms compared to glass beads biofilms and planktonic cultures, underscoring the effect of AO in mitigating resistance is growth-dependent. The better effect on biofilms compared to planktonic growth supports the role of endogenous oxidative stress in the adaptability of biofilms^3^.

It is unclear why AO has a better effect on *nfxB* mutagenesis in flow-cell biofilms than in glass beads biofilms. We can speculate that oxidative stress is relatively higher in flow cells, where there is a continuous supply of nutrients and oxygen, compared to glass beads biofilms. The MICs of CIP for isolates from planktonically evolved populations were higher than the selected isolates from evolved biofilm models, in accordance with our previous studies^25^. The AOs reduced the MIC level significantly in isolates from the CIP-AO combined treatment compared to CIP alone in both studied biofilm models but not for planktonic selected isolates.

Following previous in vitro^26^ and in vivo results^27^, no mutations in CIP target genes (*gyrA*, *gyrB*, *parA* and *parB*) were observed. Instead, mutations in efflux pumps with moderate effect on the MIC were observed. The genetic analysis of mutants from flow-cell experiments revealed mutations in *nfxB*, *nalC*, *fliG*, *flhA*, and *wspA*, which are implicated in antibiotic resistance, biofilm formation, and motility. These mutations underscore the influence of CIP and antioxidants on bacterial evolution and adaptation. The *wspA* gene plays a critical role in biofilm regulation, signal transduction, cyclic-di-GMP (c-di-GMP) signaling, virulence, and adaptation^28^. Variants in the *wspA* gene, which regulates the Wsp pathway, can lead to elevated levels of c-di-GMP, which is associated with increased biofilm formation. The mutations in *wspA* that occurred under CIP + ED and CIP + NAC treatments of biofilm have been reported in PA14 *P. aeruginosa* isolates from an in vivo model of chronic wound infection and were associated with the occurrence of the persistent phenotype of rugose small-colonies variants with elevated biofilm formation capacity^29^. The selection of *wspA* mutants in biofilms exposed to the CIP + AO combination compared to CIP alone is not entirely clear. However, it highlights the advantage these mutants have in biofilm environments and points to an unintended consequence of this treatment approach. The increased biomass observed in ED, NAC, and THU-treated flow cells biofilms (Supplementary Figure 2) warrants further investigation into the long-term implications of using antioxidants in combination with antibiotics, especially regarding biofilm-associated infections.

Additionally, the CIP-THU treatment exhibited a deletion mutation in *phzG2*, a gene involved in phenazine biosynthesis. This mutation can affect gene expression, metabolic pathways, and redox balance. Furthermore, phenazines have been shown to modify antibiotic susceptibility, indicating that mutations in these pathways could significantly impact the bacterial response to treatment^30^.

**Fig. 10:**
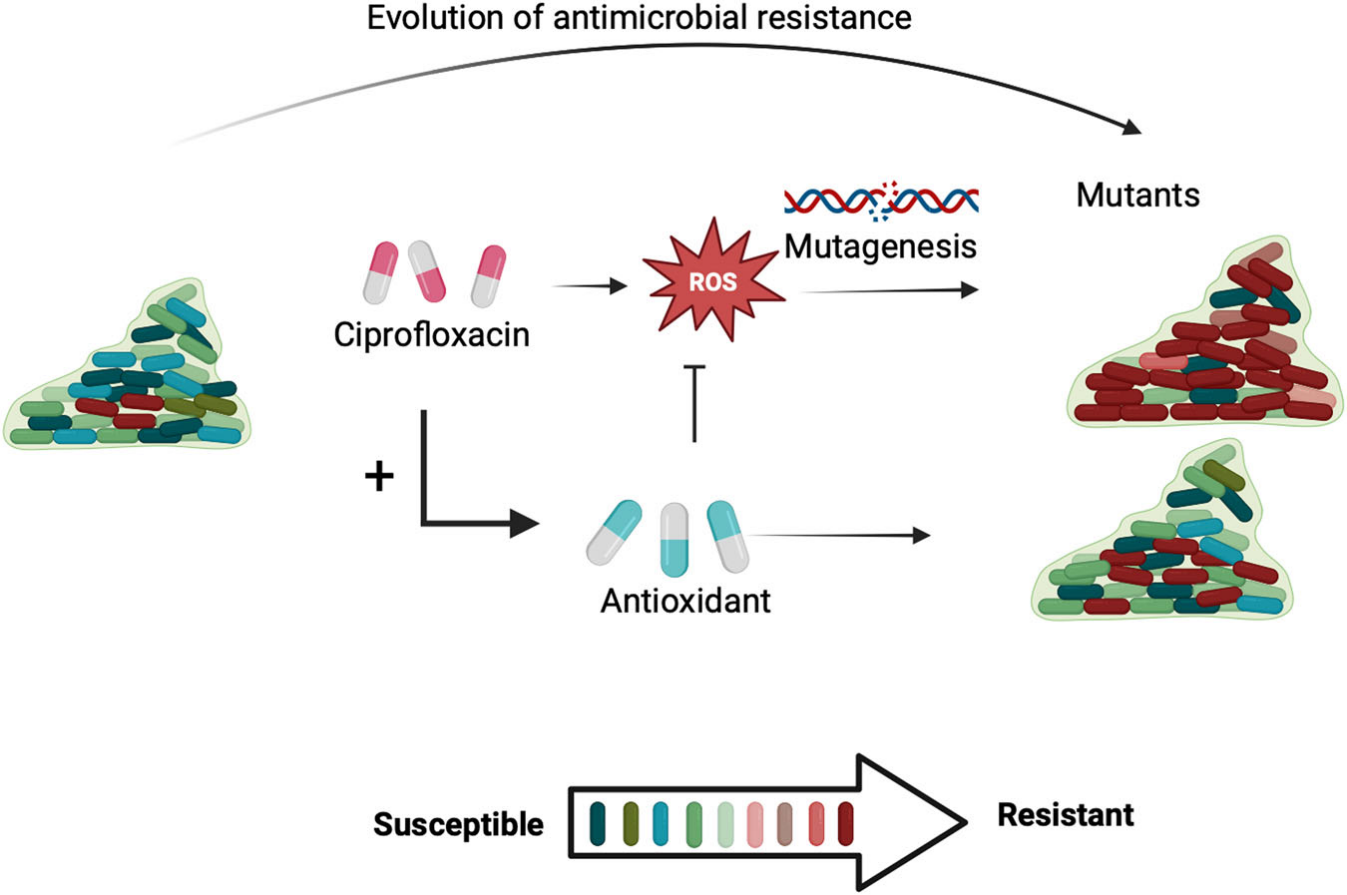
Mechanism of ciprofloxacin-induced antimicrobial resistance and the role of antioxidants. Schematic representation of the evolution of antimicrobial resistance in bacterial biofilms treated with ciprofloxacin. Exposure to ciprofloxacin leads to the production of reactive oxygen species (ROS), which induces mutagenesis, resulting in the emergence of resistant mutants. The addition of antioxidants mitigates ROS levels, thereby reducing mutagenesis and preventing the development of resistance. The gradient at the bottom illustrates the transition from a predominantly susceptible bacterial population to a more resistant one, with antioxidants acting as a protective barrier against resistance development.

The ratio of non-synonymous to synonymous mutations was calculated all over the WGS mutations revealed (Supplementary Table 1). The results show that CIP and CTRL exhibit slight positive selection, with NS/dS ratios above 1, indicating a tendency for protein-altering mutations. However, the ratio was higher with CIP, indicating a selective pressure that promotes mutations to help the bacteria adapt to survive in the antibiotic environment. In contrast, ED, NAC, and THU have NS/dS ratios below 1, reflecting purifying selection, where non-synonymous mutations are being removed to preserve protein function. Antioxidants like ED, NAC, and THU appear to stabilize the genome, reducing the occurrence of permanent mutations. This suggests that while antibiotics may drive adaptive mutations, antioxidants promote genomic stability. In conclusion, our study underscores the potential of antioxidants to mitigate CIP-induced ROS formation and mutagenesis in *P. aeruginosa* experimentally (Fig. 10). While antioxidants like ED and NAC show promise in reducing antibiotic resistance in biofilms, their impact on biofilm formation and bacterial fitness must be carefully considered. Future research should optimize antioxidant concentrations and combinations to enhance therapeutic efficacy while minimizing potential adverse effects on biofilm dynamics and bacterial virulence. This study provides a foundation for developing novel strategies to combat antibiotic resistance and improve the management of chronic biofilm-associated infections. Our in vivo model of antimicrobial resistance evolution in biofilms^27^ will allow testing of these anti-mutagenic drugs in the chronic lung infection model, revealing their effect in the complex interaction with the host immune response.

## Methods

### Bacterial strains, antibiotics, and antioxidants

In our study, we used the PAO1-*mCherry*, PAO1 with mCherry integrated at the attB site via a mini-CTX vector; the TETr marker was removed via Flp-mediated recombination. In addition to the PAO1-*mCherry-P*_*CD*_*-gfp*+ reporter strain equipped with mCherry tag and designed to feature a chromosomal transcriptional fusion between the P_*mexCD*_ promoter and the *gfp* gene^24^. The fluoroquinolone antibiotic CIP was used in this evolutionary experiment to test for the development of antimicrobial resistance. Three different antioxidants, Edaravone (3-methyl-1-phenyl-2-pyrazolin-5-one) (ED) (Merck, Denmark), N-acetyl-cysteine (NAC) (Sigma, US), and Thiourea (THU) (Sigma, US) were used to determine their ability to reduce the antimicrobial resistance to CIP. The antioxidants have been used in the following concentrations (ED 300 μM, NAC 2 mM, and THU 100 mM) after doing pilot studies to determine the concentrations that don’t have a lethal effect on the bacteria both in planktonic and biofilm growth (Supplementary Figure 6)^31^.

### Quantification of ROS by CellROX

We used a CellROX™ Green Reagent (ThermoFisher, US) detector to quantify reactive oxygen species (ROS) in live cells. The reagent is cell-permeant and has no fluorescence in the reduced state; upon oxidation, the reagent exhibits green fluorescence and remains inside the cells. Overnight cultures of the mCherry tagged PAO1 strain in LB were adjusted to OD_600_ = 0.1 and then diluted into 1000-fold in minimal ABtrace medium^32^ and treated as one of the following (CIP 0.02 µg/ml, CIP + ED 300 μM, CIP + NAC 2 mM, and CIP + THU 100 mM). In a 96-well microtiter plate (Nunc, Thermo Fisher Scientific), 100 µl of the control or treated bacterial culture was transferred and incubated at 37 °C and shaken at 225 rpm. Each treatment was represented in 5 wells (replicates), and the absorbance was measured (OD_600_ nm) using Magellan V 7.2 software every 20 min during 24 h of incubation in a pro plate reader (TECAN) with the lid on. The results for each treatment (CIP 0.02 µg/ml, CIP + ED 300 μM, CIP + NAC 2 mM, and CIP + THU 100 mM) were plotted after subtracting background values from their respective control treatments (untreated mCherry PAO1, ED 300 μM, NAC 2 mM, THU 100 mM).

### Biofilm formation in flow cells and image processing

Biofilms were cultivated within flow cells on the inner surface of attached coverslips, featuring three parallel channels. Positioned centrally between the upstream medium supply and a downstream Watson Marlow 250S peristaltic pump, the flow cells were interconnected by silicone tubing. Initially, the system underwent sterilization, wherein a 0.5% sodium hypochlorite solution flowed through. Subsequently, the system was rinsed with Milli-Q water. To acclimate the system, a continuous flow of minimal ABtrace medium supplemented with 0.3 mM glucose was maintained overnight at room temperature prior to bacterial inoculation^24^. The channels were inoculated with 200 μl of OD_600_ = 0.001 with PAO1-*mCherry*-P*CD*-*gfp* by using a sterile syringe. The flow cells were positioned upside down without the flow for 1 hour to facilitate bacterial attachment to the coverslip surface. A constant flow of medium (3 ml/h) was then maintained by using a Watson Marlow 250S peristaltic pump (Watson Marlow, UK). Replicate biofilms in 2 to 4 independent flow cells of the bacterial strain were grown for 72 hours. Then, the minimal medium was supplemented with different treatments of CIP and antioxidants, which allowed for another 72 hours of growth at room temperature. The different channels of the flow cells were optimized to be fed with a constant flow containing only one of the following (Control, CIP 0.25 µg/ml, CIP-ED 300 μM, CIP-NAC 2 mM, and CIP-THU 100 mM). Images were non-destructively imaged every 24 h (flow cells directly mounted onto the microscope stage) by using a Zeiss LSM 880 confocal laser scanning microscope (Plan-Apochromat 63/1.40 oil differential interference contrast [DIC] objective; for GFP fluorescence, excitation at 488 nm and emission range (493–587); for mCherry, fluorescence excitation at 594 nm and emission range at (586–697 nm)). Imaging was conducted 5 mm away from the inlet within the flow cell chamber. Z-stacks were created with intervals of 1 m, with the number of slices determined individually for each imaged region to encompass the entire biofilm. A minimum of three z-stacks were captured for each flow cell channel at every time point. Minimal z-stack image processing (smoothing, background subtraction, and different three-dimensional [3D] views) and the 3D photo statistics were done by using Imaris 10 software (Bitplane AG, Zurich, Switzerland). PAO1-*mCherry*-P*CD*-*gfp* biofilms were harvested by passing 1–1.5 ml of a glass bead suspension (diameter: 212–300 µm; Sigma) in saline through the flow cell channels and then collected into sterile Eppendorf tubes. Each channel’s biofilms (three bacterial populations per experiment) were isolated individually. The isolated bacterial populations were then dispersed and frozen at −80 °C for further population analysis and whole genome sequencing (WGS). An illustration describing the experimental scheme is shown in (Supplementary Fig. 7).

### Biofilm formation on glass beads

Biofilms were formed on the surface of porous glass beads (Vit- raPOR; Robu Glasfilter-geräte GmbH; diameter 4 mm, pore size 40–100 μm)^33^. The OD_600_ of an overnight culture from PAO1-*mCherry*-P*CD*-*gfp* was adjusted to 0.001 in a fresh LB medium. The beads were placed in a 50-ml Falcon tube with a filter cap containing the inoculated LB medium (1 ml/bead). The beads were incubated for 24 h at a 37 °C shaker and then washed three times by rinsing with 10 mL saline by gentle shaking and aspiration to minimize carryover of planktonic and loosely adhered bacteria on the glass surface. After washing the beads with biofilm attached were transferred into clean Falcon tubes with fresh LB medium. The tubes were designated for different treatments: Control (CIP-free), CIP treatment alone of (0.125 mg/L), or CIP treatment combined with various antioxidants—CIP + ED (300 μM), CIP + NAC (2 mM), and CIP + THU (100 mM). Subsequently, the tubes were incubated on a shaker at 37 °C for 24 h. The beads were then washed, and part of the beads (3 beads) were randomly selected from each treatment and sonicated to disperse the adhered biofilm that was then frozen in glycerol at −80 °C for further population analysis (Passage1). The rest of the washed beads were transferred to a new falcon tube with fresh LB medium with specified treatments and passed to the second passage; the steps were repeated for three consecutive passages. A model scheme is represented in (Supplementary Fig. 3).

### Planktonic evolution

Planktonic evolution experiments were conducted to determine whether antioxidants would show different effects on planktonic cells compared to biofilms in glass beads. The experiment was designed similarly to the glass beads experiment. An overnight culture from PAO1-*mCherry*-P*CD*-*gfp* was prepared from the same original colony used to start the glass beads evolution experiment. The culture was used to inoculate a 5 ml LB medium and incubated for 48 h with shaking (180 rpm) at 37 °C. The 48 h stationary phase culture was adjusted to OD_600_ 0.001 and used to inoculate five tubes of either LB tube (Control), LB with CIP 0.125 mg/L, or LB with CIP 0.125 mg/L combined with one of the three different antioxidants at similar concentrations as used in the flow cells and glass beads experiments. After 24 h of incubation at 37 °C under shaking conditions, the tubes containing various treatments underwent two washes with saline. Subsequently, 2.5 µl of OD_600_ = 0.001 was transferred from the control and treated tubes to freshly prepared tubes of control and different treatments to start a new passage of evolution. The evolution was done for three consecutive passages, and the washed cells in different passages were frozen at – 80 °C for further population analysis.

### Population analysis

Overnight cultures were prepared from bacterial populations stored frozen after being obtained from flow cells, glass beads, and planktonic evolution experiments. These cultures were serially diluted and then assessed for their resistance development by being plated on LB agar plates containing varying concentrations of CIP (0, 0.1, 0.2, and 0.5 mg/L). The ratio of the resistant subpopulation was determined by comparing the colony-forming units (CFU) of resistant colonies grown on the CIP plates to the total CFU of the population grown on CIP-free LB plates.

### Growth rates and minimal inhibitory concentrations

Bacterial isolates were retrieved from the plates with the highest concentration of CIP as identified through population analysis in the flow cells experiment. Additionally, isolates were collected from the highest CIP plates in the third passage of population analysis conducted in the glass beads and planktonic evolution experiments. Overnight cultures of the collected bacterial isolates in LB were standardized to 0.1 OD_600_ and then diluted 1000-fold. Then, 100 μl of each diluted isolate was pipetted in triplicate to individual wells of a 96-well flat-bottomed plate (Nunc, Thermo Fisher Scientific). The plate was incubated in an Infinite F200 Pro plate reader (Tecan) with the lid on, maintaining a temperature of 37 °C and shaking at 225 rpm for 24 h. Absorbance (OD_600_ nm) was measured using Magellan V 7.2 software, every 20 min during 24 h incubation. Growth rates were calculated from growth curves using the Gompertz fitting model. MICs were determined using E-test strips (bioMérieux SA, France). Five isolates were collected from the flow cell experiment, seven from the glass beads experiment, and eight from the planktonic study.

### Whole genome sequencing

An overnight culture was initiated from the harvested bacterial populations from different flow cell channels. The DNA was extracted using a DNeasy Blood & Tissue kit (Qiagen, Netherlands). DNA sequencing was performed by Illumina technology (Eurofins, Germany). The sequenced reads were checked for quality via FastQC. These reads were then aligned to the PAO1 reference genome (GenBank accession no.NC_002516) with bwa-mem, and SAMtools. The variants were called with both Freebayes and BCFtools, and the data were merged by filtering to non-synonymous variants. Finally, the variants were annotated with SnpEff.

### Statistics

Data analysis was conducted using unpaired t-tests (Prism 10, GraphPad Software, San Diego, USA). Population analysis figures were processed using R (v4.2.1). Statistical significance was set at a *P*-value ≤ 0.05. Growth curves for the various bacterial isolates were determined by fitting a Gompertz model using Prism.

## Supplementary information

Supplementary file

## Data Availability

The datasets acquired from this study are available within the publication and the attached supplemental files. The raw data files for the WGS can be accessed through the SRA project nr. PRJNA1135794.
