# Supplementary material for: The impact of antioxidant-ciprofloxacin combinations on the evolution of antibiotic resistance in Pseudomonas aeruginosa biofilms
Source: NPJ Biofilms Microbiomes. 2024 Dec 30;10:156. doi: 10.1038/s41522-024-00640-3 (PMC11685532; doi:10.1038/s41522-024-00640-3)
Supplement: Supplementary file 1 — Supplementary file [file 41522_2024_640_MOESM1_ESM.docx]

Supplementary figure 1: The growth of mCherry-tagged PAO1 (background of PAO1-mCherry-PCD-gfp) was monitored over 24 hours in the presence of CellROX, a ROS-detector, to measure green fluorescence signals. Bacterial cells were treated with Tobramycin (0.4 mg/L, black) alone or in combination with one of three antioxidants: N-acetyl-L-cysteine (Tobramycin + NAC, blue), Edaravone (Tobramycin + ED, red), or Thiourea (Tobramycin + THU, green). Consistent with the results observed with Ciprofloxacin, all antioxidant treatments significantly reduced the green fluorescence signal compared to Tobramycin alone, with *p*-values of 0.0001, 0.0407, and 0.0011, respectively.


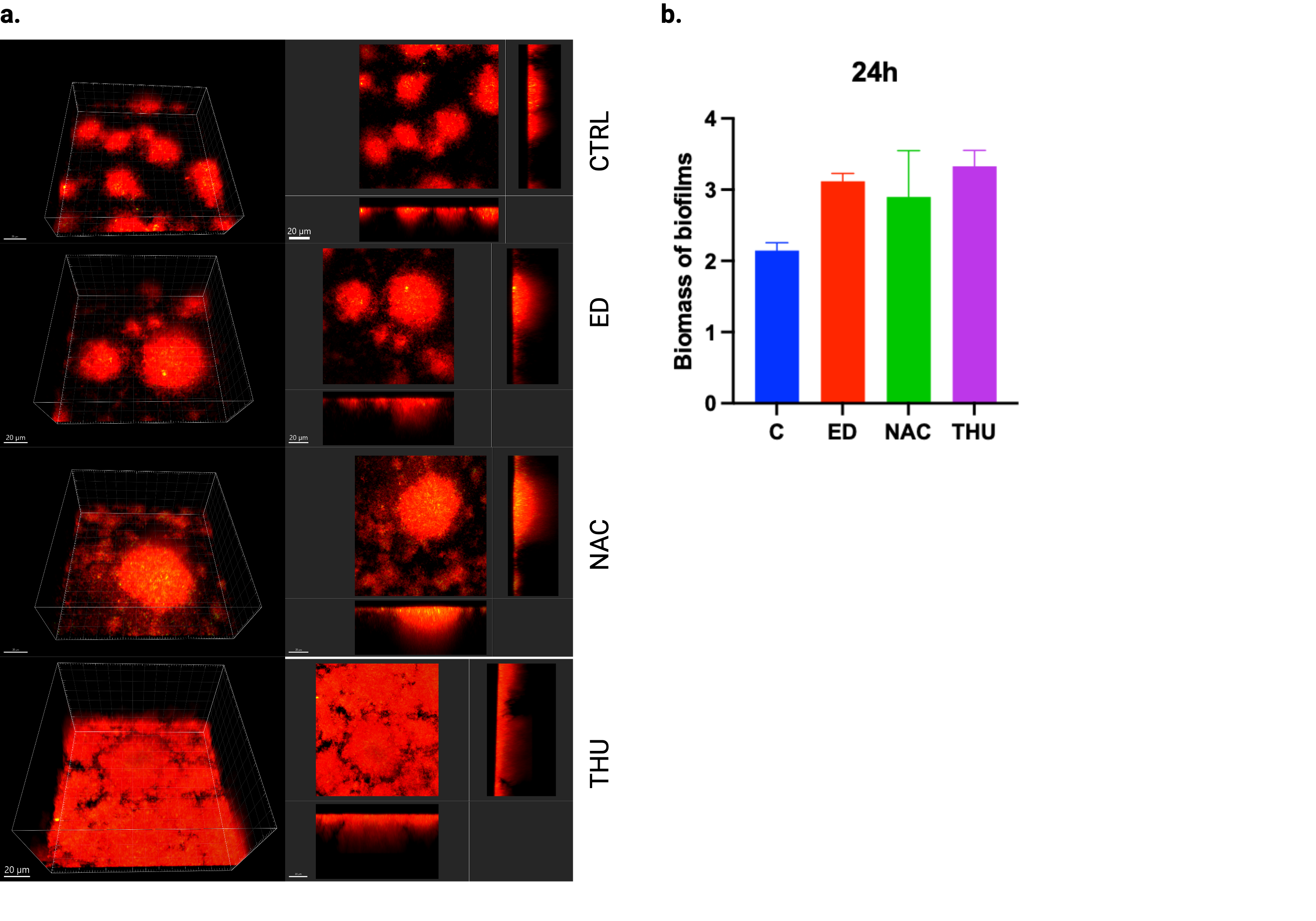


Supplementary figure 2: The effect of AOs alone on biofilm formation using the reporter strain PAO1-*mCherry*-P*CD*-*gfp*. (a) CLSM images were taken after 24 h of untreated (control) or AO- treated flow-cell biofilms with either Edaravone (ED, 300uM), N-acetyl cysteine (NAC, 2 mM), or thiourea (THU, 100 mM). Red fluorescence indicates wild-type cells constitutively expressing mCherry. Z-stacks were acquired using a Zeiss 880 microscope and processed with Imaris 10.1 software (Bitplane). The images display orthogonal 3D views of the biofilm (left panels) or sections (right panel). (b) Biomass of biofilms after 24 hours of AO treatment was quantified to assess the impact of ED, NAC, and THU compared to the untreated control.


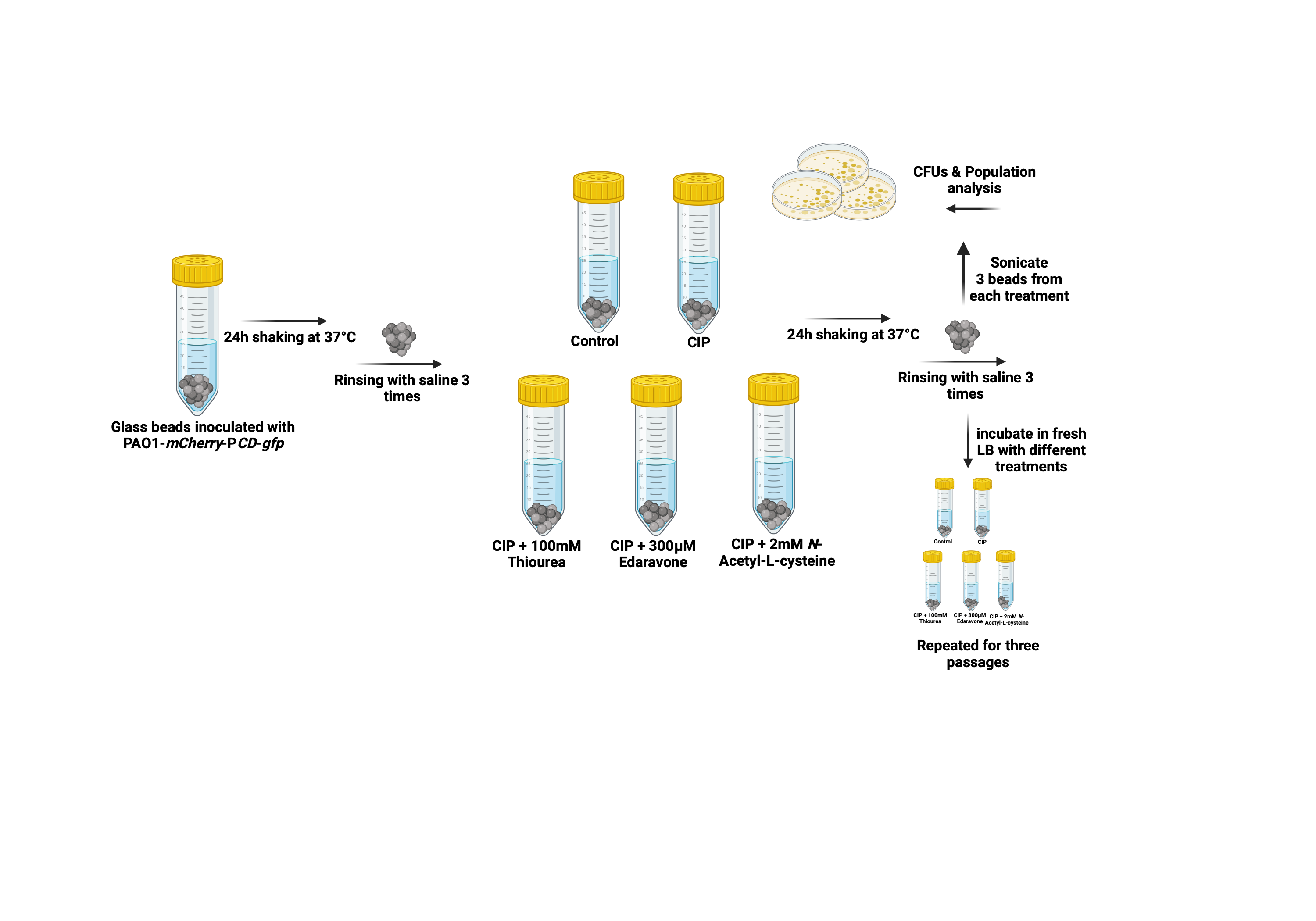


Supplementary figure 3: Experimental setup for glass beads evolution study. The experiment involved incubating inoculated LB medium with glass beads on a shaker at 37°C for 24 hours. After incubation, the beads were washed three times with saline, and fresh LB medium was added to the falcon tubes (1 mL/bead). The treatments included no treatment (control), CIP alone, or a combination of CIP with an antioxidant (CIP-AO). The tubes were then incubated for an additional 24 hours, followed by another washing step. The bacterial populations were then harvested through sonication, serially diluted, and plated for CFU counting and population analysis.


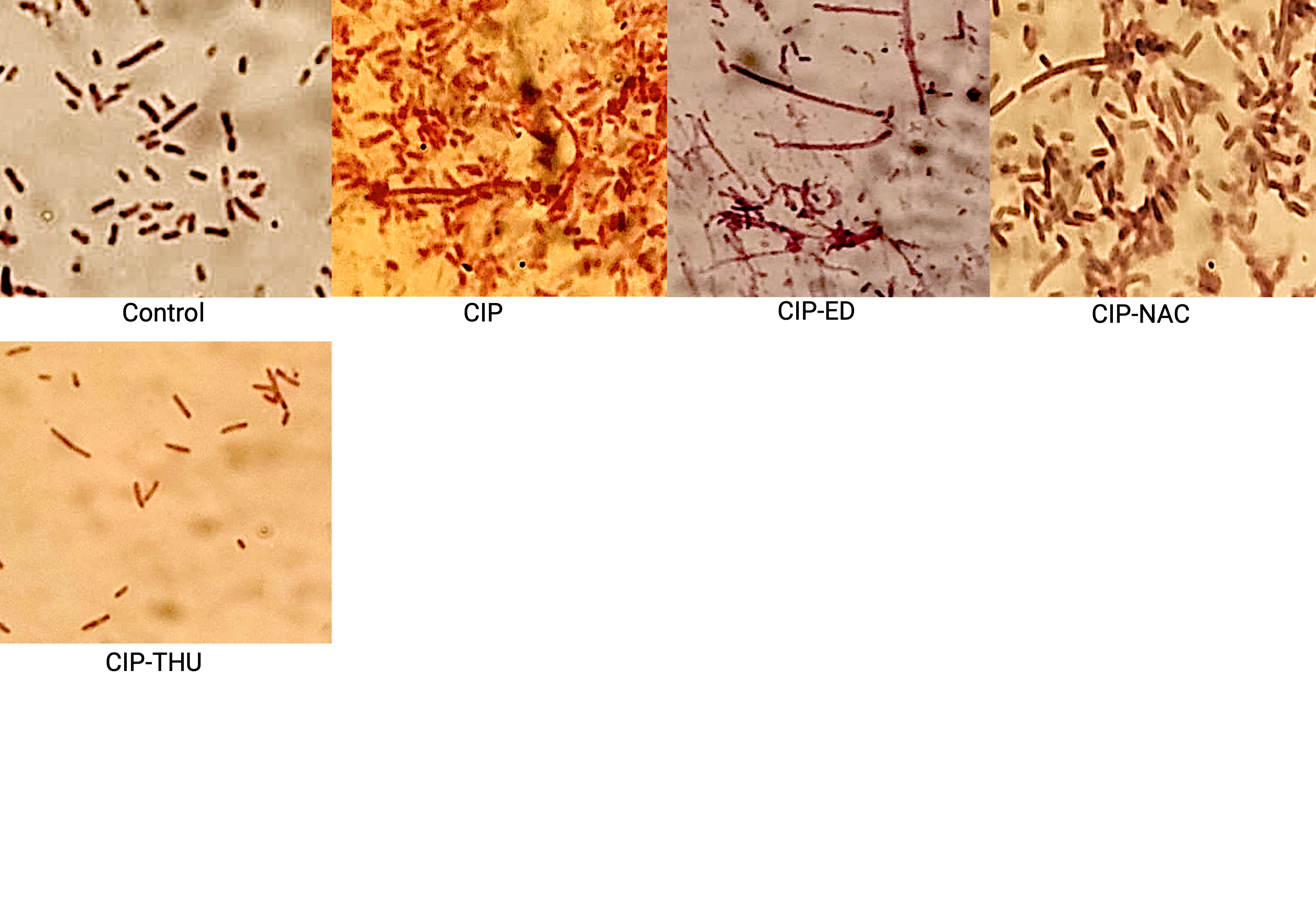


Supplementary figure 4: Gram Staining of PAO1-mCherry-PCD-gfp+ from the Final Passage of the Planktonic Evolution Experiment. Gram staining was performed on the PAO1-*mCherry-PCD-gfp+* strain collected from the final passage of the planktonic evolution experiment. Bacterial populations were exposed to various treatments, including no treatment (control), Ciprofloxacin (CIP) alone, or CIP combined with antioxidants (CIP + NAC, CIP + ED, and CIP + THU). The staining highlights the morphological differences of bacterial cells. Images were captured under a light microscope.


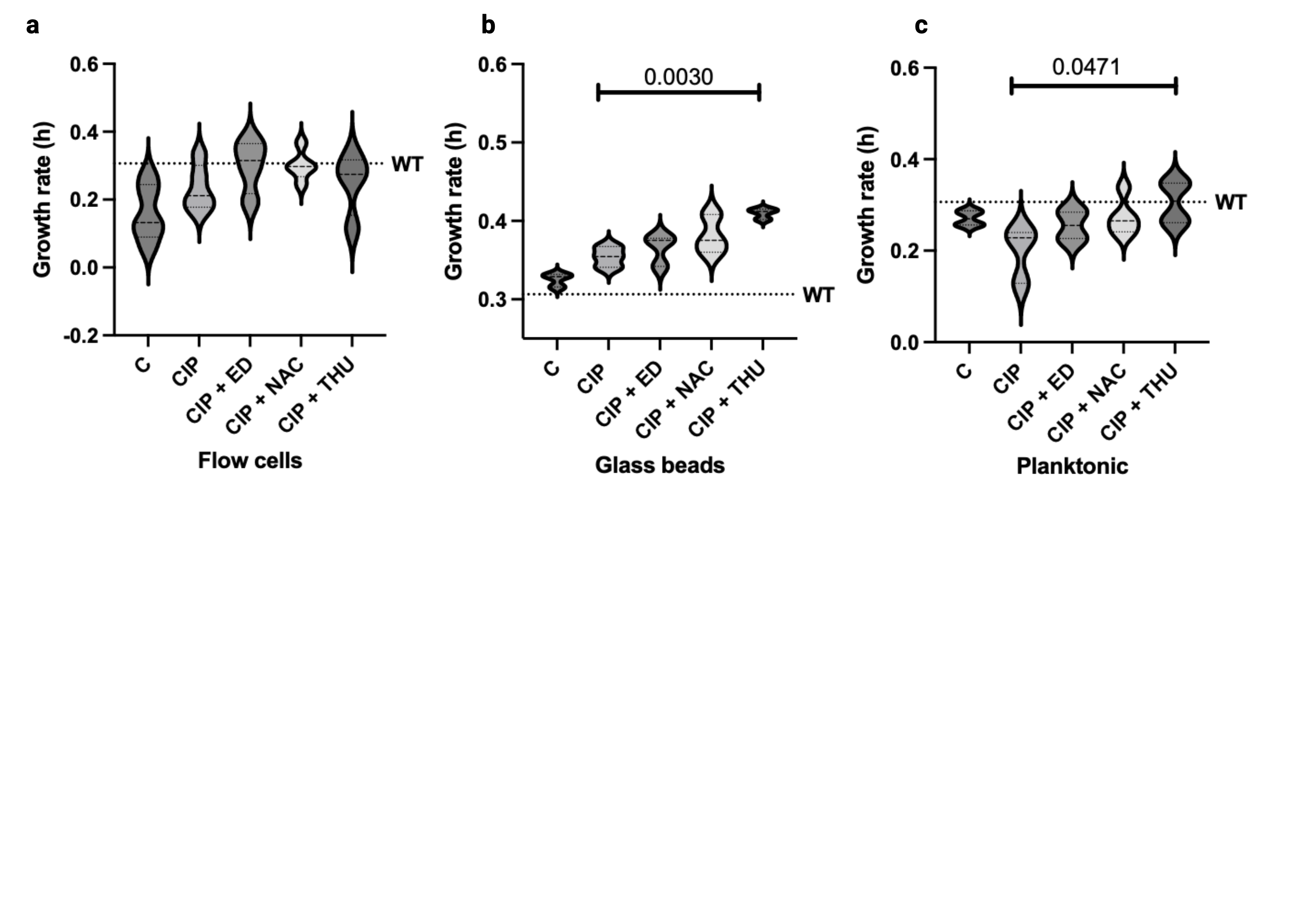


Supplementary figure 5: Growth rates calculated from growth curves of selected isolates from the highest CIP plates of different PAP in three studied evolution experiments. The plate was incubated in an Infinite F200 Pro plate reader (Tecan) with the lid on, maintaining a temperature of 37 C and shaking at 225 rpm for 24 h. Absorbance (OD_600_ nm) was measured using Magellan V 7.2 software, every 20 min during 24 h incubation. Growth rates were calculated from growth curves using the Gompertz fitting model.


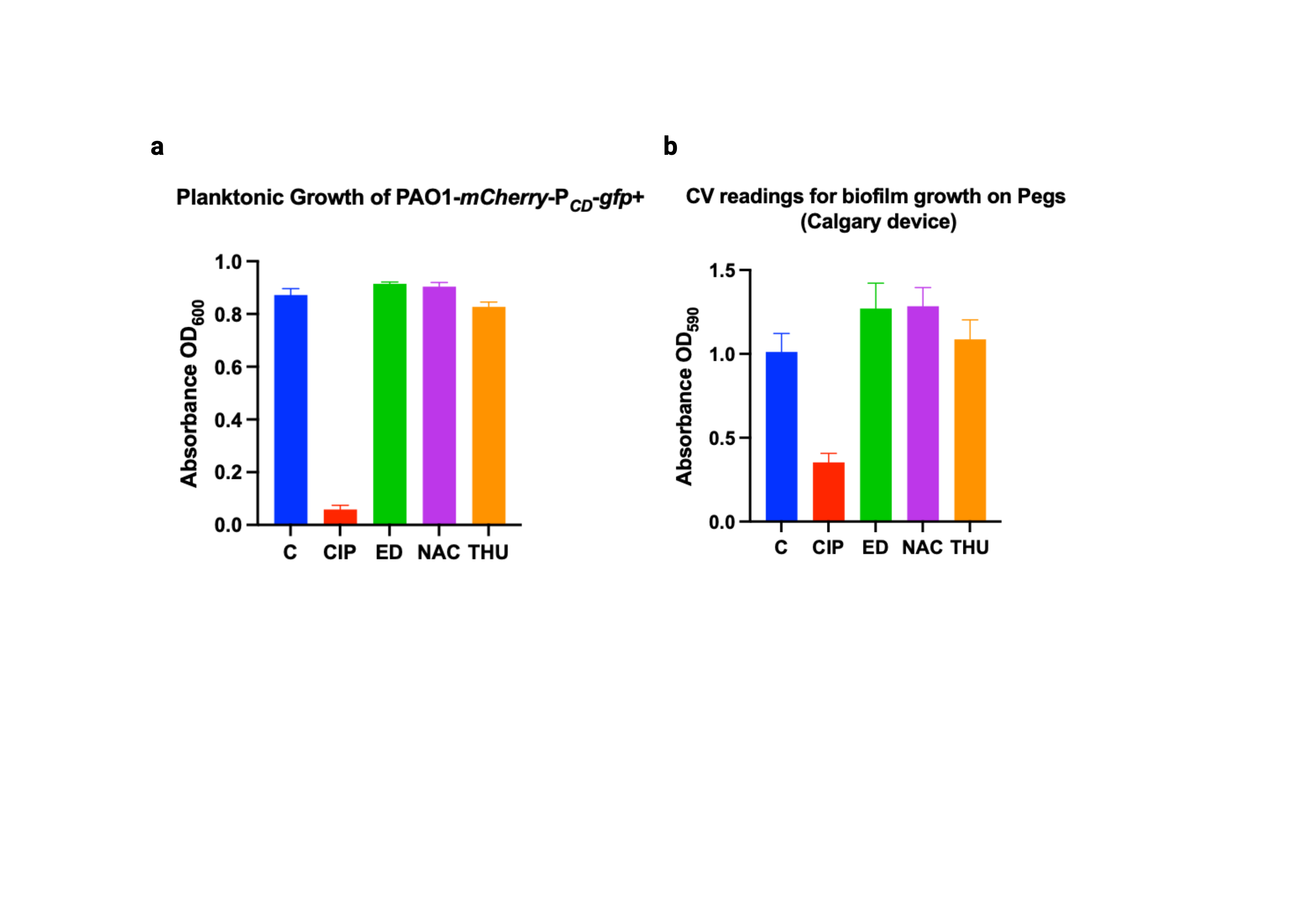


Supplementary figure 6: Antibacterial Activity of Ciprofloxacin (CIP) and Antioxidants in Planktonic and Biofilm Growth. (a) Planktonic Growth in LB Medium: The antibacterial activity of CIP (0.02 µg/mL) and the different antioxidants edaravone (ED, 300 µM), N-acetyl cysteine (NAC, 2 mM), and thiourea (THU, 100 mM) was assessed in planktonic cultures of PAO1-*mCherry-PCD-gfp.* (b) Biofilm Formation in the Calgary Device: The antibacterial effects of CIP (0.25 µg/mL) and the antioxidants ED, NAC, and THU were tested on biofilms formed on the plastic peg lids of the Calgary biofilm device. Biofilms were grown for 24 hours and then exposed to the treatments for an additional 24 hours. Following treatment, biofilms were washed and biomass was visualized using crystal violet staining (1).


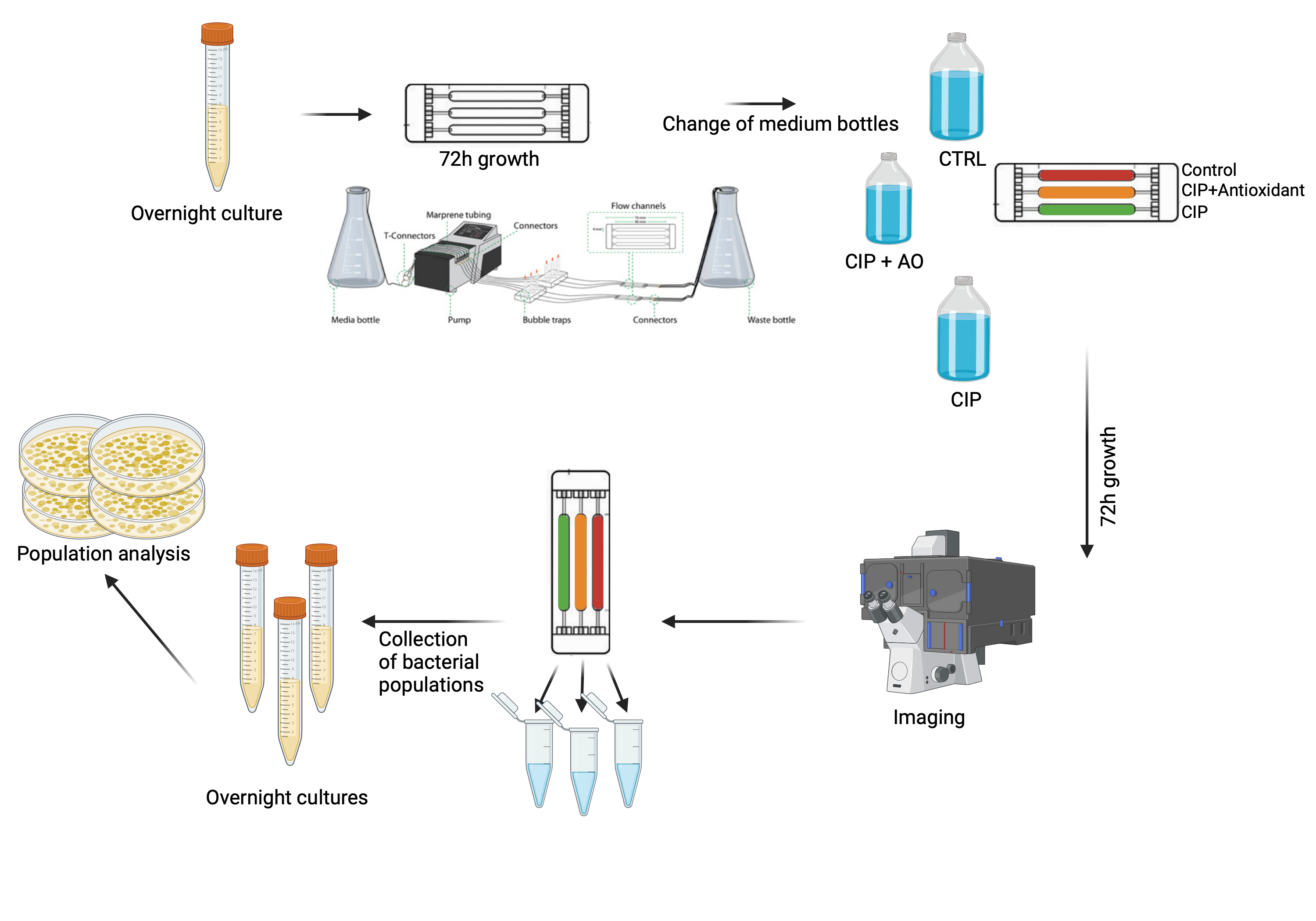


Supplementary figure 7: An illustration of the different steps of the flow cells experiment followed in the current study. An overnight culture of PAO1-*mCherry-PCD-gfp* was diluted to an OD₆₀₀ of 0.001, and 200 µl of the culture was used to inoculate each flow cell channel. The flow cells were positioned upside down without medium flow for 1 hour to allow bacterial attachment to the coverslip surface. After the attachment phase, a continuous flow of minimal ABtrace medium was initiated at a rate of 3 ml/h. During the first 72 hours, all flow cells were fed from the same ABtrace medium bottle to allow biofilm growth. For the subsequent 72 hours of treatment, individual small bottles containing specific treatment conditions were connected to each flow cell. Confocal laser scanning microscopy (Zeiss LSM 880) was used to capture biofilm images at 24, 48, and 72 hours post-treatment. Biofilms were then harvested by passing 1–1.5 ml of a sterile saline suspension containing glass beads through each channel. The harvested biofilm populations were collected into sterile Eppendorf tubes, with each channel's biofilm (three populations per experiment) kept separate. The harvested biofilm populations were used to start new overnight cultures, which were subsequently analyzed for population characteristics.

|  | NonSyn no. | Syn no. | NS/dS ratio |
| --- | --- | --- | --- |
| CTRL | 14 | 12 | 1,17 |
| CIP | 14 | 11 | 1,27 |
| ED | 14 | 17 | 0,82 |
| NAC | 12 | 15 | 0,80 |
| THU | 14 | 16 | 0,88 |

Supplementary Table 1: The table summarizes the number of non-synonymous (NonSyn) and synonymous (Syn) mutations observed, along with the calculated NS/dS ratio, for different experimental treatments. The treatments include untreated control (CTRL), ciprofloxacin alone (CIP), and combinations of ciprofloxacin with antioxidants edaravone (ED), N-acetyl cysteine (NAC), and thiourea (THU). The NS/dS ratio represents the proportion of non-synonymous mutations to synonymous mutations, indicating the potential selective pressure under each condition.

1. Ciofu O, Rojo-Molinero E, Macià MD, Oliver A. Antibiotic treatment of biofilm infections. Apmis. 2017;125(4):304-19.
